# Thinking about thinking: changes in first-year medical students’ metacognition and its relation to performance

**DOI:** 10.3402/meo.v20.27561

**Published:** 2015-08-26

**Authors:** Wei Han Hong, Jamunarani Vadivelu, Esther Gnanamalar Sarojini Daniel, Joong Hiong Sim

**Affiliations:** 1 Medical Education and Research Development Unit (MERDU), Faculty of Medicine, University of Malaya, Kuala Lumpur, Malaysia; 2 Department of Mathematics and Science, Faculty of Education, University of Malaya, Kuala Lumpur, Malaysia

**Keywords:** metacognitive skills, selection tool, medical students

## Abstract

**Background:**

Studies have shown the importance of metacognition in medical education. Metacognitive skills consist of two dimensions: knowledge of metacognition and regulation of metacognition.

**Aim:**

This study hypothesizes that the knowledge and regulation of metacognition is significantly different at the beginning and end of the academic year, and a correlation exists between the two dimensions of metacognitive skills with academic performance.

**Methods:**

The Metacognitive Skills Inventory comprising 52 Likert-scale items was administered to 159 first-year medical students at the University of Malaya. Students’ year-end results were used to measure their academic performance.

**Results:**

A paired sample *t*-test indicated no significant difference for knowledge of metacognition at the beginning and end of the academic year. A paired sample *t*-test revealed significant difference for regulation of metacognition at the beginning and end of the academic year. A very strong correlation was found between the two dimensions of metacognition. The correlation between knowledge and regulation of metacognition with students’ academic result was moderate.

**Conclusions:**

The improvement in students’ metacognitive regulation and the moderate correlation between knowledge and regulation of metacognition with academic performance at the end of the academic year indicate the probable positive influence of the teaching and learning activities in the medical program.

The concept of metacognition was first introduced in the 1970s by John Flavell who described it as the knowledge concerning one's own cognitive processes. Metacognition was also referred to as learners’ automatic awareness of their own knowledge and their ability to understand, control, and manipulate their own cognitive processes (1). Past research has shown that students who possess metacognitive skills are able to perform better in their studies and are high achievers (2). Kentridge and Heywood (3) and Reder and Schunn (4) put forward that metacognitive skills need not operate in a person's conscious awareness. Metacognition is when one takes control of one's own learning, plans and selects strategies, and monitors and evaluates the progress of his or her learning (5). Metacognitive skills are also referred to as higher-order thinking skills, which are needed to help individuals to become more adaptable, flexible, and able to cope in the context of a rapidly evolving information society. There are two dimensions of metacognition identified: metacognitive knowledge and metacognitive regulation, which were the focus of the present study.

## Metacognitive skills in medical school

It is important to emphasize and acknowledge the importance of metacognitive skills in the teaching and learning process, especially in a medical school. According to Gonullu and Artar (6), metacognition in the context of medical schools is a concept that attempts to capture the essence of adapting to changes and uncertainties. Hence, students cannot rely entirely on their teachers’ teaching all the time but must be able to plan and utilize the knowledge in a wide variety of tasks. Medical students must also be prepared to cope with the changes and uncertainties with their evolving understanding – which is essential during medical practice. Students who have the habit of following instructions blindly will suffer from ineffective cognitive performance in intellectual tasks. In the case of medicine, doctors are expected to perform excellently and be independent lifelong learners who are able to continuously assess the outcome of their actions to build new knowledge (7). Medical students who have low metacognitive skills will face problems as they will be unable to determine the difficulty of the tasks, plan their actions, monitor their own performance, or use information and graphical representations. Past research has shown that undergraduate medical students’ independent learning in terms of monitoring and guiding their own learning process does affect their achievement (8).

## Metacognitive knowledge

Education theorists believe that the development of metacognitive knowledge begins at a young age and continues through adolescence (9). Metacognitive knowledge encompasses declarative knowledge, procedural knowledge, and conditional knowledge (10, 11). Students who are high achievers in reading, writing, mathematics, and science also exhibit higher levels of metacognitive knowledge and have developed greater abilities in self-regulation (12). The research findings of Hoseinzadeh and Shoghi (13) also noted that students’ metacognitive knowledge will have an effect on their abilities to improve their academic performance.

Medical students and practitioners are expected to be knowledgeable and extremely effective and efficient despite their limited time and mental resources at a particular moment in diagnosing and making complex decisions (14). In medical schools, one of the most common stressors identified is the pressure of academics with an obligation to succeed (15). The level of metacognitive knowledge in medical students is an essential continuous process which reflects on their performance. Research carried out has shown that problem-based learning (PBL) has been introduced to medical students to help medical students apply more of the acquired knowledge (16).

## Metacognitive regulation

Metacognitive regulation consists of three essential skills: planning, monitoring, and evaluation (9, 11, 17). In the context of medical students, metacognitive regulation encourages better use of the basic medical knowledge in nurturing clinical judgment, critical thinking, and reflective practice (18). It also refers to a reflective approach to problem solving that involves visualizing a problem to examine and reflect on the thinking process (19).

Although Brown (10) argued that metacognitive regulation may be an unconscious act, researchers have found that metacognitive regulation requires a conscientious effort of thinking and does affect the performance of students (9, 20). Hence, the level of metacognitive regulation in medical students is important in enabling students to plan, monitor, and evaluate themselves in order to be better learners and cope with the hectic syllabus in the MBBS program.

The aim of the present study was to investigate the two dimensions of metacognitive skills of first-year medical students at the University of Malaya who followed the revised University of Malaya Medical Programme (UMMP). The hypotheses tested in this study were as follows: 1) knowledge of metacognition is significantly different at the beginning and end of the academic year; 2) regulation of metacognition is significantly different at the beginning and end of the academic year; and 3) a correlation exists between the two dimensions of metacognitive skills with academic performance.

## Methodology

This study involved the participation of the first cohort of first-year medical students for the revised UMMP.

### Participation

All 179 first-year medical students enrolled in the revised UMMP were invited to participate and complete the survey. Students who choose to enter the University of Malaya must first obtain perfect scores irrespective of whether they had sat for the different pre-university studies offered in Malaysia such as *Sijil Tinggi Pelajaran Malaysia* or the Matriculation and University of Malaya Foundation Studies. The participation of students was, however, not mandatory. Thus, 159 (88.83%) first-year UMMP medical students participated in the research. The participation involved a 30-minute session for answering the Metacognitive Skills Inventory (MSI).

### Ethics approval

Ethics approval was granted by the Medical Ethics Committee, University Malaya Medical Centre (Ethics committee/IRB reference number 1010.50).

### Study design and data collection

#### Metacognitive Skills Inventory

The MSI was adapted from the Metacognitive Awareness Inventory (MAI) designed by Schraw and Dennison (20). The MAI has been used in the field of education for research purposes (8, 20–24). In medical education, Turan and Demirel (25) carried out a study on metacognition with preclinical students, where the MAI was used and one-to-one interviews were done. Even though the items of the inventory developed by Schraw and Dennison (20) were taken as the basis for the development of this scale, a different scale was developed and was improved in this study. In another research, a questionnaire was designed for measuring attitudes towards group and individual learning, alongside with the MAI (26).

Eichbaum (27) described the metacognitive approach to the medical humanities and how it is designed to develop students as eager learners and flexible thinkers, together with their capacity for cognitive and emotional monitoring and regulation. Stansfield et al. (28) in a study on metacognition revealed that metacognitive effort showed the largest decline over time and affects their clinical empathy attitudes. The emergence of metacognitive effort in the clinical years suggests empathy may appear lesser for students after clinical exposure and may have resulted in much of the observed decline in clinical empathy attitudes.

There are a total of 52 six-point Likert-scale items in this inventory which were adapted according to the context of the medical field. In the MSI, the statements comprised two categories of metacognition: knowledge of metacognition and regulation of metacognition (20). The knowledge of metacognition consists of statements on declarative knowledge, procedural knowledge, and conditional knowledge (29). The regulation of metacognition covers a wide perspective which involves planning or goal setting, organizing and managing information, and monitoring in which students assess their learning process and strategies used. The regulation of metacognition also involves the debugging process which engages students to use strategies to correct errors or reflect on mistakes done, whereas the evaluation process is where students analyze their performance and weigh the effectiveness of the strategies used.

The medical students’ year-end results were used as a measurement of their academic performance. The year-end results encompassed 30% of Required Summative Assessment and 70% of Barrier Assessment. The students sat for a written examination, also known as the Barrier Assessment, at the end of the academic year which assessed the core knowledge content.

### Statistical analysis

Paired sample *t*-tests were used to determine whether there was a significant difference in 1) knowledge of metacognition at the beginning and end of the academic year, and 2) regulation of metacognition at the beginning and end of the academic year. Pearson correlation coefficients were computed to study correlations between the two dimensions of metacognitive skills and students’ academic performance.

## Results

Table 1 shows the metacognitive skills scores of the medical students. At the beginning of the academic year, a mean score of 241.97 was obtained, whereas the overall score ranged from a minimum of 183 to a maximum of 292, with a standard deviation of 25.21. A mean score of 237.67 was obtained at the end of the academic year, whereas the overall score ranged from a minimum of 175 to a maximum of 300, with a standard deviation of 28.14.

**Table 1:** Paired sample *t*-tests of metacognitive skills scores at the beginning and at the end of the academic year (*n=*159)

	Paired differences				
				
Pairs	Mean	SD	*t*	df	Level of significance
Pair 1					
Knowledge (beginning) – Knowledge (end)	1.30	10.32	1.59	158	0.114
Pair 2					
Regulation (beginning) – Regulation (end)	−3.76	20.05	−2.36^a^	158	0.019
Pair 3					
Metacognitive skills (beginning) – Metacognitive skills (end)	4.30	27.65	1.96	158	0.052

a *t*-value is significant at the 0.05 level.

### Dimensions of metacognitive skills at the beginning and at the end of academic year

The paired sample *t*-tests showed there was no significant difference in the scores for the dimension of knowledge of metacognition scores in first-year medical students at the beginning of the academic year (*M*=77.18, SD=10.79) and at the end of the academic year (*M*=75.87, SD=9.81) conditions [*t*(158)=1.59, *p*=0.114 as shown in Table 1]. These results suggest that first-year students’ metacognitive knowledge has not differed much after going through the first-year teaching and learning process. In the first year, students learn basic sciences whereby much of the content is repetitive science knowledge with more depth. The basic sciences taught build upon subjects learned in pre-university level and follow the spiral curriculum.

The paired sample *t*-tests showed there was a significant difference in the scores for the dimension of regulation of metacognition scores in first-year medical students at the beginning of the academic year (*M*=161.29, SD=19.27) and at the end of the academic year (*M*=165.04, SD=16.11) conditions; *t*(158)=−2.36, *p*=0.019 as shown in Table 1. These results suggest that that first-year students’ metacognitive regulation does change after a year. This shows that there is an increase in students’ level in regulation of metacognition after going through the first academic year. The results suggest that medical students in their first year showed better ability to plan, monitor, and evaluate their own thoughts after going through one academic year. The findings appear similar to Maudsley and Strivens (18) who thought regulation of metacognition encourages better use of the basic medical knowledge in nurturing clinical judgment, critical thinking, and reflective practice.

This change could be due to ward rounds introduced at this early stage and PBL sessions which were aimed to encourage the students to manage and relate what has been taught in the classroom with their clinical exposures. These findings also support the findings of the research carried out by Dochy et al. (16) which showed that PBL has been introduced to medical students to help them apply more of the acquired knowledge. In previous studies, ward rounds have been shown to be important in effective interpretation of information learned in the formal classroom which can enhance the metacognitive monitoring processes of the students (30). The students learn and start to see the real-world scenario which could lead them into deeper thought processing for more meaningful learning. This finding also matches the research by Kincannon et al. (31) which indicated that understanding and regulating one's own cognitive processes enables the person to monitor, direct, and control information.

The paired sample *t*-test showed there was no significant difference in the scores for the total score of metacognitive skills scores in first-year medical students at the beginning of the academic year (*M*=241.97, SD=25.21) and at the end of the academic year (*M*=237.67, SD=28.14) conditions; *t*(158)=1.96, *p*=0.052 as shown on Table 1. The researchers suggest that because there is no significant difference in the level of metacognitive skills at the beginning and at the end of the academic year of the first-year medical students, medical students’ performances were somewhat predictable even from the beginning of the academic year.

### Metacognitive skills and performance

At the beginning of the academic year, the relationship between knowledge of metacognition (*r*=0.38), regulation of metacognition (*r*=0.34), and metacognitive skills (*r=*0.37) with students’ academic results revealed a weak correlation as shown in Table 2. This indicates that medical students may have acquired the knowledge but were not able to utilize the knowledge and keep up with the pace to set goals, plan, and evaluate themselves accordingly. This result suggested that medical teachers should assist the medical students and doctors in developing the skills they need to effectively cope with the challenges presented by new environments to transform the new encounter from a threat to a learning opportunity (32).

**Table 2:** Correlation between the dimensions of metacognitive skills and academic results at the beginning the academic year (*n*=159)

		Academic results
Knowledge	Pearson correlation	0.375**
	Sig. (2-tailed)	0.000
Regulation	Pearson correlation	0.335**
	Sig. (2-tailed)	0.000
Metacognitive skills	Pearson correlation	0.373**
	Sig. (2-tailed)	0.000

** Correlation is significant at the 0.01 level (2-tailed).

At the end of the academic year, the correlation of the knowledge of metacognition (*r*=0.35), regulation of metacognition (*r*=0.50), and metacognitive skills (*r*=0.47) and the academic results revealed statistical significance at the 0.05 level. The results showed only a weak correlation between knowledge of metacognition and year-end results, whereas regulation of metacognition and metacognitive skills showed moderate correlations with their year-end results respectively as shown in Table 3.

**Table 3:** Correlation between the dimensions of metacognitive skills and academic results at the end of the academic year (*n*=159)

		Academic results
Knowledge	Pearson correlation	0.353**
	Sig. (2-tailed)	0.000
Regulation	Pearson correlation	0.504**
	Sig. (2-tailed)	0.000
Metacognitive skills	Pearson correlation	0.472**
	Sig. (2-tailed)	0.000

** Correlation is significant at the 0.01 level (2-tailed).

Strength of the correlation between regulation of metacognition and the academic results was statistically higher than the knowledge of metacognition. Once again, this difference attributes to the different environment introduced in the UMMP curriculum. The findings of this correlation oppose Schraw and Dennison (20) who found the knowledge of cognition factor of the MAI was related to higher test performance, and the regulation of cognition factor of the MAI was not. This scenario may be true in the context of education in general, but medical students in the UMMP curriculum are exposed to ward rounds at an earlier stage. This appears similar to the findings of Eva and Regehr (14) who stated that medical students are expected to be knowledgeable and efficient in making decisions when they practice later.

## Limitations of the study and suggestions for further research

This study involves only one cohort of medical students which is gathered only from one medical school. These students were first-year medical students who were still in the transition period between pre-university to university environment when the study was conducted. The year-end result which was used to measure students’ academic performance comprised only the written examination component.

Based on the study conducted, further research can be conducted using different cohorts of medical students in different medical schools to further validate the MSI as a tool for selection.

## Conclusions

Both the dimensions of metacognitive skills, which are knowledge and regulation of metacognition, showed significant difference before and after the academic year. The strong correlation between the knowledge and regulation of metacognition and students’ academic results showed that the MSI was somewhat indicative of students’ academic performance. This is because metacognitive skills are emerging essential components for doctors, and these medical students will potentially be at the front line making real decision when limited resources and time constraint apply.
